# The Spatiotemporal Evolution Analysis of Ecosystem Pattern in Wenchuan (Magnitude 8.0) Earthquake Disaster Area, China

**DOI:** 10.3390/ijerph18052490

**Published:** 2021-03-03

**Authors:** Bing Zhang, Peng Hou, Hai-tao Xu, Yan-hong Zhao, Jun-jun Bai, Xian-lin Liu

**Affiliations:** 1 College of Resource Environment and Tourism, Capital Normal University, Beijing 100048, China; zhangbing_1991as@163.com (B.Z.); xht0807@sina.com (H.-t.X.); liuxl@cae.cn (X.-l.L.); 2 Satellite Environment Center of the Ministry of Ecology and Environment, Beijing 100094, China; skdzyh@163.com (Y.-h.Z.); 18851776166@163.com (J.-j.B.); 3 College of Geoscience and Surveying Engineering, China University of Mining & Technology, Beijing 100083, China; 4 Chinese Research Academy of Environmental Sciences, Beijing 100012, China

**Keywords:** ecological system, landscape pattern, grey correlation, coupling coordination, entropy weight, TOPSIS

## Abstract

The ecological system is the basis of human survival and global environmental protection. In the process of development, countries will pay close attention to the changing state of the ecosystem. Taking the ecosystem pattern as the research object, a three-layer analysis method was proposed. The transfer matrix and landscape index were used as the first layer to analyze the basic changes. Grey correlation, range-coupling coordination and relative priority were used as the second layer to analyze the reasons of the change. The interval-entropy weight, TOPSIS (Technique for Order Preference by Similarity to an Ideal Solution), was used as the third layer to evaluate the quality of the change. The ten counties in the worst-hit areas of the Wenchuan earthquake were analyzed from different angles, with county region, intensity zone and ecosystem as the objects, and the following results were obtained: (1) Taking Mianzhu City as an example, from 2000 to 2010 and 2018, the conversion ratio of forest, grassland and farmland is 54.24, 59.19, 17.21, 20.06, 37.39 and 52.86%, which were distributed in the north, central and southern parts, respectively. (2) Taking the ninth intensity zone as an example, the forest landscape fragmentation increased, disturbance decreased, and species diversity increased. There is a high influence and restriction relationship between ecosystem and landscape pattern in the total landscape area change. Additionally, the relationship between them tends to develop in a benign way. As of 2018, it is in the change state of moderate imbalance-ecosystem lag. (3) Taking the county ecosystem change as an example, urban type is the best in the counties of ecosystem change, of which Shifang is the best and Pingwu is the worst. The results show that this method can effectively compare and analyze the changes in the multi-regional ecosystem pattern, which has the characteristics of universality and can also be applied to the research of ecosystem pattern change in special regions.

## 1. Introduction

An earthquake is a kind of natural phenomenon during which the earth’s crust releases energy. It has particularity, complexity and unpredictability. At the same time, the timely, efficient, accurate acquisition, integration and reasonable assessment of the disaster ecological situation after the occurrence of earthquake disasters are of great significance to disaster relief, reconstruction and later recovery in the earthquake area. However, the ecological [1,2,3,4] status of earthquake areas is closely related to human property and biological survival, so it is necessary and urgent to explore the changes in the ecosystem [5,6,7,8] caused by earthquake. Ecosystem pattern [9,10,11] refers to the representation and embodiment of regional ecosystem and its landscape pattern [12,13] characteristics on the spatial scale. In terms of ecosystem, it is reflected in the composition classification and its spatial heterogeneity. In terms of landscape pattern, it is reflected in the random distribution and characteristic effect of patches of different classifications on landscape types [14,15,16]. From the perspective of spatial distribution and change, the survival of organisms depends on the conditions, so the ecosystem pattern is the basis of the research of ecosystem. Major earthquakes have a far-reaching impact on the ecosystem. The world’s top 10 historical earthquakes are all above magnitude 8.5, one of which occurred in China, and three of the top 10 historical earthquakes in China were above magnitude 8. At present, research on ecosystem pattern mostly focus on the purpose, most of which focuses on the analysis of regional ecosystem security, ecosystem pattern change or ecosystem characteristics. There is little research on the evaluation of ecological pattern under special natural conditions, such as earthquakes. How to analyze and evaluate the dynamic pattern of regional ecosystems with scientific means from the perspective of earthquake is not only a scientific research topic with special significance, but also a social topic with great practical significance. Therefore, this paper decided to take the Wenchuan earthquake [17] as an example to carry out a long-term temporal and spatial analysis of ecosystem pattern evolution in order to explore a theoretical method suitable for the study of ecosystem pattern evolution in earthquake-stricken areas. 

**Figure 1:**
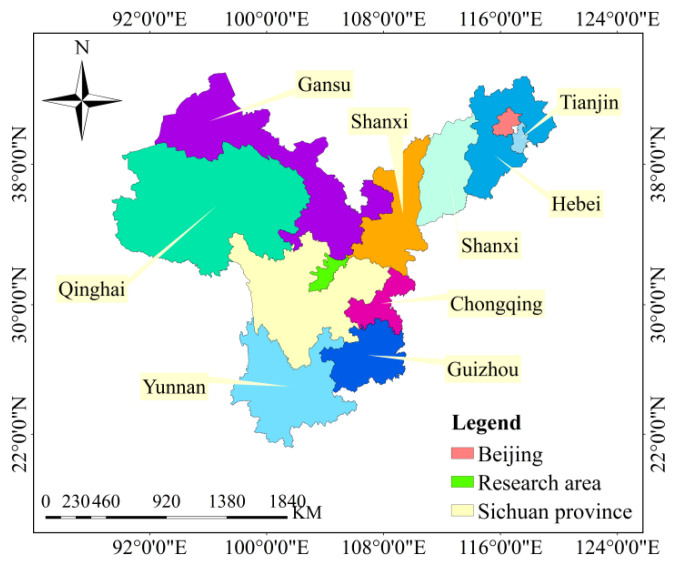
Map of the research area location.

Most of the current research methods on the change in ecosystem pattern tend to classify the ecosystem first and analyze the changes and transitions of its composition. Then, the landscape change characteristics of the ecosystem were analyzed through the landscape index, research on the relationship between ecosystem services and landscape patterns or research on the relationship between ecosystem and regional economic development. Moreover, most of the studies focus on a specific region, so there is a lack of comparison of multi-region universality of research methods. In this paper, based on the current analysis theory, the experiment and the integration of cross-disciplinary analysis experience, the ecosystem pattern is divided into three levels: ecosystem type and landscape pattern index, correlation and coupling coordination and evolution results. The main theories are as follows: Firstly, the change in ecosystem pattern depends on ecosystem type and landscape index, so the change in ecosystem basic composition is analyzed by the change and shift of ecosystem composition type and landscape pattern index. Second, there is a certain interdependent relationship between ecosystem composition and landscape characteristics change, which makes their changes show a coupling effect. Therefore, the exact status of ecosystem pattern change can be analyzed effectively through correlation and coupling. In the third layer, the comparison of changes before and after the earthquake is the standard to evaluate the restoration, so the ecosystem restoration results are analyzed by the amount of changes in the ecosystem. Ten counties in the worst-hit area of Wenchuan earthquake in China were selected as the study area, and the analysis was carried out according to the three-layer analysis theory. In this way, the practicability of the method can be verified through multi-regional analysis, and the universality of the method can be verified through the research of the change in the ecological pattern in this special region. The location of the research area is shown in Figure 1 below. The paper consists of five parts. The Introduction introduces the current research status, the research purpose, significance, theory, method, expected results and the structure of the paper. The Overview of the Study Area mainly introduces the basic situation of the region of ecosystem pattern analysis. The Research Methods mainly involve the analytical methods and standards used. The Results section shows the calculation results and analysis. The Conclusion and Discussion mainly discuss the results of the examples and further discuss the research theory.

## 2. Overview of the Study Area

**Figure 2:**
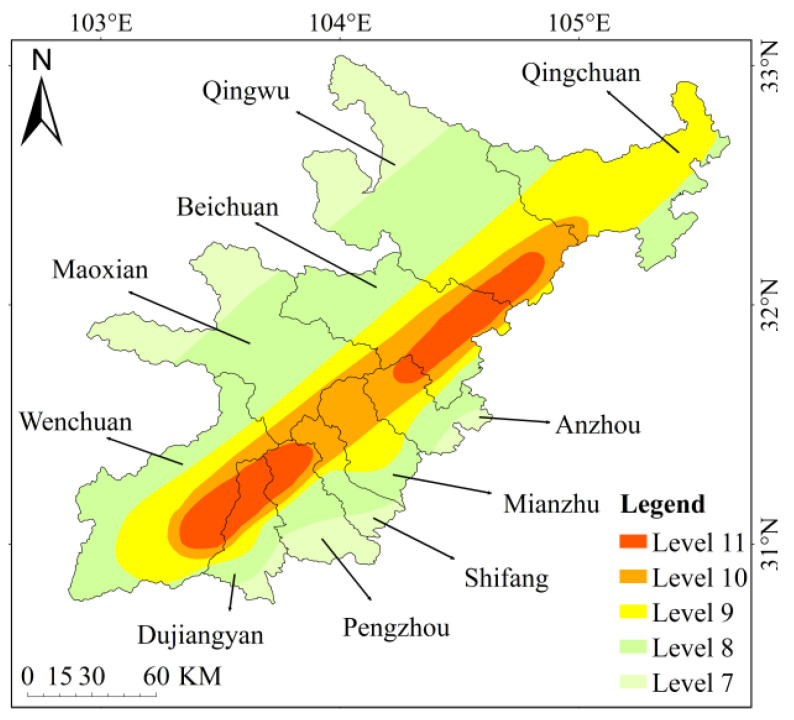
Seismic intensity division map of the research area.

**Figure 3:**
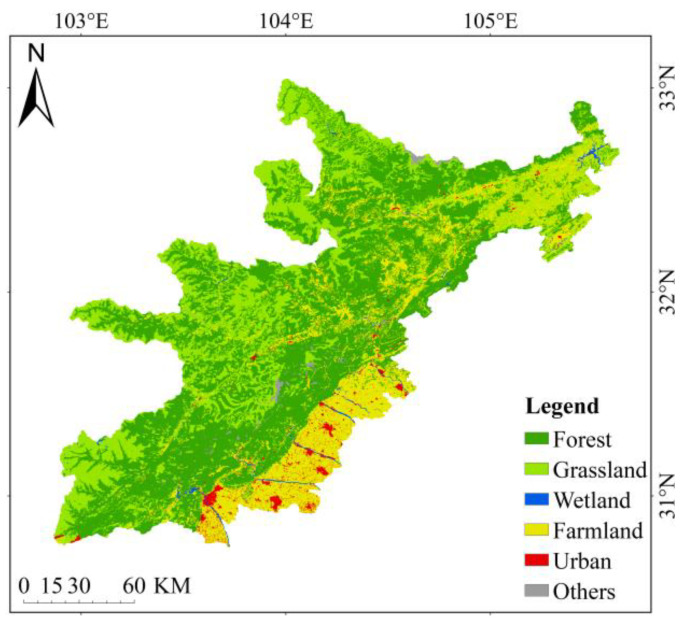
Map of ecosystem composition in 2018.

An earthquake occurred in Wenchuan County, Sichuan Province, China at 2 PM, 28 min and 4 s on 12 May 2008 Beijing Time. With a surface wave magnitude of 8.0 Ms and a moment magnitude of 8.3 Mw, the earthquake intensity reached 11 degrees, the damage area exceeded 100,000 km, the affected population reached 5 million and the economic loss was 845.1 billion yuan. It was the most destructive earthquake since the founding of China. The research location is between 102 and 105 degrees east longitude and between 30 and 33 degrees north latitude. According to the county region, it is divided into ten units: Wenchuan, Maoxian, Beichuan, Pingwu, Qingchuan, Anzhou, Mianzhu, Shifang, Pengzhou and Dujiangyan. According to the intensity zone level, it is divided into five units of level 7, level 8, level 9, level 10 and level 11. The specific division is shown in Figure 2 below. According to the ecological characteristics, composition mode and ecological uses of the land, the ecological system in the research area was divided into six categories: namely, forest, grassland, wetland, farmland, urban and other unused in a scientific and reasonable way that is suitable for human production and living. The present status of ecosystem composition and distribution in the research area is shown in Figure 3 below.

## 3. The Research Methods

Wenchuan earthquake is the deadliest earthquake after Tangshan earthquake (the deadliest earthquake in the world’s history in the 20th century). It is of great practical and social significance to choose Wenchuan earthquake as the research object. In addition, it has been more than 10 years since the earthquake occurred, and periodic studies are of certain value to judge the changes and recovery degree before and after the earthquake. At the same time, choosing the earthquake area as the analysis area can also provide scientific reference for the impact of similar situations in the future. Finally, through the multi-level division and multi-unit comparison of the research area, it can not only judge and evaluate the impact of earthquake on the ecosystem pattern from different perspectives, but also scientifically analyze the influencing factors of the evolution of ecological pattern. Therefore, ten counties in the worst-hit areas of Wenchuan earthquake were selected as the analysis area, and the period from 2000 to 2018 was selected as the time period. The changes in ecosystem pattern before and after the earthquake were analyzed by stages and regions in terms of time and space.

This paper proposes a three-layer analysis method for the evolution of ecosystem patterns in earthquake areas. Ecosystem type and landscape pattern change are taken as the first layer, which is mainly analyzed by statistical and transfer matrix [18]. The second layer is the relationship between ecosystem types and landscape pattern index, which is mainly analyzed by the grey correlation method [19], coupling coordination method and relative priority degree. The third layer is the quality of evolution results, which is mainly analyzed by the TOPSIS(Technique for Order Preference by Similarity to an Ideal Solution) method [20]. The applicability and universality of the method can be verified through the comparative analysis of ten units in the county region, five units in the intensity zone and six categories of ecosystem through the three-layer analysis method. Due to the large number of experimental results, one object was selected, respectively, from the county, intensity zone and ecosystem to display the results of the first, second and third layer analysis methods. 

### 3.1. Changes in Ecosystem Types

Transfer matrix is often used for land use spatial change analysis, and the core idea of it is the Markov model [21]. Five types, including forest, grassland, wetland, farmland and urban, were selected to carry out quantitative calculation with Markov model, so as to obtain the amount, direction and ratio of mutual transformation among different types under seismic disturbance. When it comes to statistical calculations, since there are six classes objectively, all types are involved in the calculation. Only select indicators are used in the calculation of evaluation and comparison. Basic data came from the Data Center for Resources and Environmental Sciences, Chinese Academy of Sciences. Basic data mainly include remote sensing classified images of 2000, 2005, 2010, 2015 and 2018.

The transfer matrix is shown in Equation (1) as follows:

(1) $$A_{ij}=\left[\begin{array}{ccccc} A_{11} & A_{12} & A_{13} & \cdots & A_{1n}\\ A_{21} & A_{22} & A_{23} & \cdots & A_{2n}\\ A_{31} & A_{32} & A_{33} & \cdots & A_{33}\\ \cdots & \cdots & \cdots & \cdots & \cdots \\ A_{n1} & A_{n2} & A_{n3} & \cdots & A_{nn} \end{array}\right]$$

In the formula, A represents the sum of the areas of a particular type in an ecosystem. Additionally, $A_{ij}$ represents a change from type *i* to type *j*, and the transition matrix is usually presented in an Excel spreadsheet.

### 3.2. Change in Landscape Pattern

#### 3.2.1. Landscape Pattern Index

Landscape index can reflect the heterogeneity of landscape change in landscape pattern analysis well. Six landscape pattern indices were selected and divided into four categories according to their ecological significance and application: integrity index, fragmentation index, disturbance index and species diversity index. The corresponding index results were obtained at class level by Fragstat4.2 software. Based on this, the response relationship between landscape pattern and ecosystem change is analyzed, as shown in Table 1 below.

#### 3.2.2. Grey Relational Degree Method

Grey correlation analysis [22,23,24] is generally used in multi-factor statistical analysis, and its application has been widely verified in determining the correlation between factors. The correlation degree between factors is reflected through sample changes. In the case of a small number of samples, the grey correlation method has some advantages in analyzing the correlation between landscape index and ecosystem type change.

(2) $$\xi_{m,n}=\frac{\frac{min}{m}\frac{min}{n}\left|X_{ij}-Y_{ij}\right|+\rho \frac{\max}{m}\frac{\max}{n}\left|X_{ij}-X_{ij}\right|}{\left|X_{ij}-Y_{ij}\right|+\rho \frac{\max}{m}\frac{\max}{n}\left|X_{ij}-X_{ij}\right|}$$

In the formula, $\xi_{m,n}$ represents the correlation coefficient between indexes, $\frac{min}{m}\frac{min}{n}\left|X_{ij}-Y_{ij}\right|$ and $\frac{\max}{m}\frac{\max}{n}\left|X_{ij}-X_{ij}\right|$ represent the minimum and maximum absolute difference, respectively. ρ represents the resolution coefficient, in the ranges of 0 ~ 1 and generally takes a number of 0.5.

(3) $$R_{m,n}=\frac{1}{N}\sum_{m,n=1}^{N}\xi_{m,n}$$

In the formula, $R_{m,n}$ denotes the correlation degree, which ranges from 0 to 1. A larger number means it is more relevant and there is more coupling. Among them, *m*,*n* = 1,2…*N*, *N* is the number of objects.

The data are processed dimensionless. Taking the total area of landscape type as the reference value and landscape index and ecosystem type as the object, the grey correlation method was used to analyze the correlation between landscape change and landscape index and ecosystem type. Symmetric classification is used to highlight high correlation above, low correlation below and weakened medium correlation for seismic ecosystem. The correlation degree is divided into six levels in order to obtain the main correlation, as shown in Table 2 below.

### 3.3. Change in Landscape Pattern

Coupling coordination method is often used to measure the coupling cooperative relationship between objects. Coupling degree refers to the mutual influence and correlation between systems, reflecting the degree of restriction between them. Coupling coordination degree [25,26] reflects the benign degree of coupling action and the quality of coordination. There is interdependence and interaction between ecosystem composition and landscape pattern change. The coordinated development of ecosystem and landscape pattern can be obtained by measuring the coupling coordination relationship between ecosystem and landscape pattern.

(4) $$C_{1}(U_{1}\times U_{2}\times \cdot \cdot \cdot U_{n})=n\times {\left[\frac{U_{1}\times U_{2}\times \cdot \cdot \cdot U_{n}}{(U_{1}+U_{2}+\cdot \cdot \cdot U_{n}{)}^{n}}\right]}^{\frac{1}{n}}$$

In the formula, *i*,*j* = 1,2,…,*n*, *C_n_* denotes the coupling degree, then *C*_2_ is:

(5) $$C_{2}=2\times {\left[\frac{U_{1}\times U_{2}}{\left(U_{1}+U_{2})(U_{1}+U_{2}\right)}\right]}^{\frac{1}{2}}$$

(6) $$U_{n}=\sum_{i=1}^{n}Y_{i}W_{j}$$

In the formula, *U_n_* represents the subsystem value, which is generally guaranteed to be between 0 and 1. *Y_i_* represents the dimensionless index, and *W_j_* represents the weight.

In order to avoid the situation that the coordination number of index is low and the difference is not large, but the calculation result is high, the model needs to be modified, as follows:

(7) $$D=\sqrt{C\times T}$$

(8) $$T=\beta_{1}\times U_{1}+\beta_{2}\times U_{2}+\cdot \cdot \cdot \beta_{n}\times U_{n}$$

In the formula, *T* represents the coordination index, *D* represents the coordination degree, and β represents the weight. 

The range method is used to standardize the indicators. The weight was obtained by entropy weight method, and the comprehensive evaluation value of ecosystem and landscape pattern was calculated according to the weight. Additionally, then, the comprehensive value is normalized. Finally, the coupling coordination relationship between them is measured by the coupling coordination method, and the benign degree of the coupling effect is obtained. In this way, it reflects the merits and demerits of changes in coordination. Secondly, the relative priority model [27,28] of ecosystem was established based on the normalized comprehensive evaluation value. The lead and lag of the ecosystem relative to the landscape pattern were measured to explain the reasons for the change in the coupling coordination. This research suggests that when the relative priority is between (0, 1), ecosystem change lags behind landscape pattern. When the priority is between1 and 100, they change synchronously. When the priority is greater than 100, the ecosystem changes are faster than the landscape pattern. Among them, the ecosystem indexes are the five selected ecosystem types. *NP*, *PD*, *LPI* and *MPS* are selected as landscape pattern indexes, and the classification standard of coordination level was shown in Table 3 below.

### 3.4. Assessment of Ecosystem Change

Entropy weight method [29,30,31,32] can effectively avoid the interference of human subjective factors to the evaluation index. The TOPSIS method [33,34] in a multi-objective decision evaluation can compare the evaluation object with the obtained “positive and negative ideal distance” to get the “relative proximity”. The results are based on the relative proximity. It has good practicability in evaluating the merits and demerits of multi-index changes. Interval method is applied to process the index. The weight of each type is obtained by entropy weight method. Then, a new weighting matrix is obtained by recalculating according to the weight. Finally, the TOPSIS method was used to sort the results. The comparative advantages and disadvantages of ecosystem changes in different regions can be obtained through interval-entropy weight, TOPSIS.

The calculation formula of the optimal scheme $Q_{j}^{+}\;$ and the worst scheme $Q_{j}^{-}$ in TOPSIS is as follows:

(9) $$Q_{j}^{+}\;=(\max r_{i1},\max r_{i2},\cdots ,\max r_{im})$$

(10) $$Q_{j}^{-}\;=(\min r_{i1},\min r_{i2},\cdots ,\min r_{im})$$

Calculate the Euclidean distance $d_{i}^{+}$ and $d_{i}^{-}$ between each evaluation object and the two schemes:

(11) $$d_{i}^{+}=\sqrt{\sum_{j=1}^{m}(Q_{j}^{+}-r_{ij}{)}^{2}\;},d_{i}^{-}=\sqrt{\sum_{j=1}^{m}(Q_{j}^{-}-r_{ij}{)}^{2}\;}$$

The relative proximity calculation formula is as follows:

(12) $$C_{i}=\frac{d_{i}^{-}}{d_{i}^{+}+d_{i}^{-}}$$

The result $C_{i}$ is a number between 0 and 1, representing the change in region *i*. The larger the number is, the better the condition is; otherwise, the worse it is. Combined with the Euclidean distance value, the relative proximity is calculated and sorted, and the evaluation results are obtained.

## 4. Results Analysis

### 4.1. Analysis of Change Difference in Different Counties

By comparing the degree of change among counties, it is found that Dujiangyan and Wenchuan have the best conditions until 2018, and the conditions of them are basically the same. In Dujiangyan, forest and farmland are the main components of the ecosystem, and the amount of farmland change is the largest and has been in a decreasing trend. Forest and grassland were the main components of the ecosystem in Wenchuan, which increased or decreased to varying degrees during the period. Mianzhu and Anzhou are the worst. In Mianzhu, forest and farmland are the main components of the ecosystem, and farmland has been in a decreasing trend during this period. In the ecosystem composition of Anzhou, the area of farmland and forest is the largest, and the change proportion of farmland is smaller than that of forest. The worst Mianzhu city was selected as the main object of example. 

**Figure 4:**
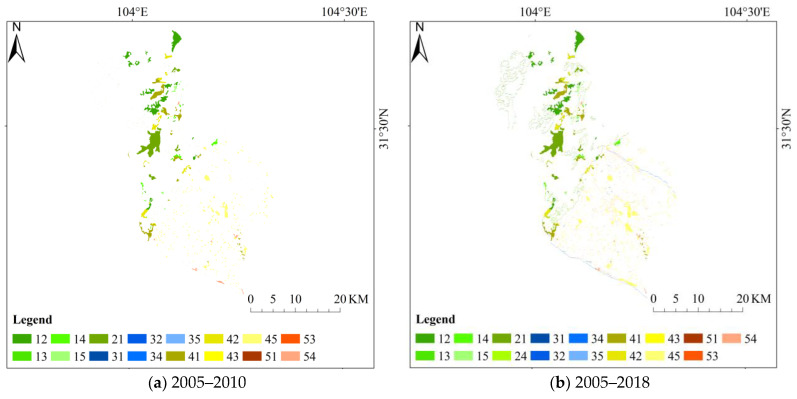
Spatial changes in ecosystems in Mianzhu City from 2005 to 2018. Note: The legend numbers represent each type in the ecosystem. Among them, 1–5 represent forest, grassland, wetland, farmland and urban. Each legend number represents a change in the type from left to right. For example, the number 12 represents the change from forest to grassland.

According to the analysis of spatial distribution and transfer matrix, the spatial distribution of ecological types in Mianzhu from 2005 to 2018, the transfer proportion of forest by 2010 and 2018 was 54.24 and 59.19%, respectively, which was in an increasing state. Combined with the spatial distribution, it can be seen that the changes occurred during the earthquake were mainly distributed in the north of Mianzhu. The proportion of grassland turning out in the two periods was 17.21 and 20.06%, respectively, which was increasing, mainly distributed in the middle part. The conversion ratio of farmland in the two periods was 37.39 and 52.86%, respectively, which was in an increasing state, mainly distributed in the southern area of Mianzhu. The spatial changes in ecological types in Mianzhu from 2005 to 2018 are shown in Figure 4 below. According to the spatial transfer changes in the overall ecological types in Mianzhu, the types of changes occurred during the earthquake were all over the entire district. In addition, the earthquake made the types of changes have a clear boundary, indicating that the distribution of various ecological types in Mianzhu is relatively concentrated and evenly distributed. The transformation matrix of ecological types in Mianzhu from 2005 to 2018 is shown in Table 4 below.

### 4.2. Analysis of Variation Difference of Different Intensity Zones

By comparing the variation degree of intensity zone, it is found that until 2018, the best intensity zone is level 9. Among them, the proportion of forest is the largest, and urban has been increasing from the perspective of changes. The worst intensity zone is level 8. Forests and grasslands accounted for a large proportion of them. From the change situation, forest and farmland had been in a decreasing trend with a similar range of changes. The nine order intensity zone with the best condition was selected as the main object of example. 

From 2000 to 2018, forest accounted for the largest proportion of ecosystem types in the level 9 intensity zone before the earthquake. Therefore, forest was selected as the response object of landscape pattern characteristic change. After the earthquake, *CA* and *MPS* were reduced by 6146.37 and 102.32 hm^2^, respectively. It shows that the forest condition becomes worse after the earthquake and the integrity decreases. *NP* and *PD* increased by 68 and 0.0013, respectively. It shows that the fragmentation of landscape pattern increases, which is consistent with the decrease in integrity. *PLAND* decreased by 0.111% compared with that before the earthquake, indicating that the influence of forest in landscape pattern was reduced, and the disturbance force was reduced. *LPI* was on an increasing trend with an increase of 0.0153%. This indicates an increase in species diversity. According to calculation, the highest comprehensive correlation degree of landscape total area change is *LPI*, and the lowest correlation degree is urban. Judging from the overall results, there is a high influence and restriction relationship. Among them, *LPI* has the strongest correlation, and urban has the strongest constraint. The correlation results are shown in Table 5 below.

It can be seen from the coupling coordination results that the overall coupling after the earthquake is higher than that before the earthquake, indicating that the coupling relationship is better. The overall coordination is in an increasing trend, indicating that the coordination becomes better and is in a benign development trend after the earthquake. The reason is that the comprehensive value of the ecosystem decreased and increased after the earthquake, while the comprehensive value of landscape pattern changed little. As a result, although the change in the ecosystem lagged behind the landscape pattern, the change distance between them gradually decreased, making the development tend to balance. The coupling coordination results of level 9 are shown in Table 6 below.

### 4.3. Analysis of the Change Difference of Different Ecosystem Types

Comparing the changes in different ecosystem types, the results show that until 2018, among the ecosystem types of county, the best is urban and the worst is farmland. The sorting is shown in Table 7 below. The urban with the best condition was selected as the main object of example. The proportion of urban in each county increased compared with that before the earthquake. Qingchuan had the best farmland condition and Dujiangyan had the worst. In the intensity zone, the best ecosystem type condition is urban and the worst is forest. The urban condition in each intensity zone was better than that before the earthquake. The best forest condition is level 8, and the worst is level 10.

**Figure 5:**
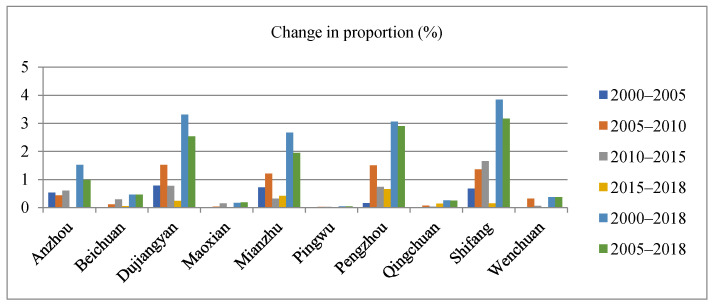
Statistics on urban characteristics change from 2000 to 2018 (county).

The urban area of each county is relatively small. Based on the situation of different regions, Dujiangyan, Mianzhu and Shifang are relatively large, while Pingwu and Wenchuan are relatively small. The analysis time was divided into three stages. The first stage was from 2000 to 2018, and the changes in each stage were compared in different time periods. The discontinuity points were 2005, 2010 and 2015. The regions with the largest changes in the four stages were Dujiangyan with an increase of 0.78 and 1.52%, Shifang with an increase of 1.66% and Pengzhou with an increase of 0.65%. The second stage is the overall long-term time series comparison from 2000 to 2018. Dujiangyan, Pengzhou and Shifang had large changes in area, increasing by 3.32, 3.06 and 3.84%, respectively. Pingwu had a small change with an increase of 0.04%. The third stage is the comparison before and after the earthquake to judge the recovery degree, which is from 2005 to 2018. Dujiangyan, Mianzhu, Pengzhou and Shifang had better conditions after the earthquake, which had changed to the level before the earthquake in 2010. The urban area of Anzhou, Beichuan and Wenchuan increased little compared with that before the earthquake. The changes in Maoxian, Pingwu and Qingchuan were similar to the same status before the earthquake, and the urban area increased by 0.03, 0.02 and 0.06%. In terms of overall recovery, Shifang had the best situation from 2005 to 2018, with an increase of 3.17%, and Pingwu was the worst, with an increase of 0.04%. The changes in urban characteristics at the county level from 2000 to 2018 are shown in Figure 5 below.

## 5. Discussion

First of all, the three-layer analysis method proposed in this paper has certain applicability and universality from the application process and calculation results. At the same time, compared with other similar studies, the results obtained by this method are more ideal and can reach the expected goal. However, from the perspective of analysis, there are still some defects: the spatial and temporal evolution of ecosystem pattern is based on certain external conditions. An earthquake is a sudden natural disaster that causes change, but it also involves other conditions. Other factors such as human development, species migration and natural changes may also contribute to change. Therefore, it may be necessary to further analyze the driving forces causing the change and verify the response relationship between the driving forces and the change. However, these works may need further scrutiny because of the lack of actual field and other geographic information to support further analysis.

Secondly, as a way to understand the basis of ecosystem and analyze the evolution of ecosystem, ecosystem pattern research has made a series of achievements, but there is still a lot of work to be done: First, the ecosystem pattern reflects the objective evolution law of the ecosystem, which determines that the work is carried out in a long-term and multi-sectoral way. Second, the ecosystem is a complex system, with complex information sources, numerous objects, fuzzy technical levels and disciplinary boundaries. Accurate research requires the overall integration of other factors and resources, such as society, environment, geography, etc.

Finally, the evolution of ecosystem pattern indicates the possibility of future changes, and we can try to predict and analyze the future change trend and possible evolution results. Therefore, it is believed that the driving force of change can be analyzed from the combination of multiple data in the depth research. It can also make a prediction from the perspective of space and try to use the method in this paper to analyze the future trend of change combined with the prediction results.

## 6. Conclusions

In this paper, a three-layer analysis method is proposed based on the current status and needs of ecosystem research in earthquake-stricken areas. The Wenchuan earthquake in Sichuan Province, China, is taken as an example for practical research, and the spatiotemporal evolution characteristics of the research area are discussed by this method. The conclusion is as follows:

From the perspective of methods:

(1) The three-layer analysis method based on the current study of ecosystem pattern change can be used to analyze and compare the merits and demerits of ecosystem pattern change in multi-region and special region, which has certain application and popularization value.
(2) From the results of spatial analysis, landscape index analysis, grey correlation, coupling coordination, relative priority and multi-region comparative evaluation, the practicability and universality of this method can be verified.

From a theoretical perspective:

(1) The process and result of ecosystem pattern change can be reflected scientifically through the analysis steps from shallow to deep.
(2) The analysis results verify the feasibility of the theory, and the operability of the method makes the theory achieve the purpose of research.
(3) The combination of theory and method has been verified in practice, which reflects the practical significance and social value of the research.

## Figures and Tables

**Table 1:** Landscape pattern index classification.

**Landscape Pattern and Ecosystem Response**	**Integrity Index**	Total(Class) Area (CA)
		Mean Patch Area (MPS)
	**Fragmentation Index**	Number of Patches (NP)
		Patch Density (PD)
	**Disturbance Index**	Percentage of Landscape (PLAND)
	**Species Diversity Index**	Largest Patch Index (LPI)

**Table 2:** Grey correlation index grade.

Correlation	0~0.25	0.25~0.45	0.45~0.55	0.55~0.65	0.65~0.85	0.85~1.00
**Correlation Types**	Very low	Low	Medium low	Medium high	High	Very high

**Table 3:** Standard for grading coupling coordination degree.

D Value	Coordination Level	Coupling Coordination Degree	D Value	Coordination Level	Coupling Coordination Degree
(0.0~0.1)	1	Extreme imbalance	[0.5~0.6)	6	Barely coordination
[0.1~0.2)	2	Serious imbalance	[0.6~0.7)	7	Primary coordination
[0.2~0.3)	3	Moderate imbalance	[0.7~0.8)	8	Moderate coordination
[0.3~0.4)	4	Mild imbalance	[0.8~0.9)	9	Good coordination
[0.4~0.5)	5	Near imbalance	[0.9~1.0)	10	Best coordination

**Table 4:** The transfer matrix of ecosystem types in Mianzhu city from 2000 to 2018(Km^2^).

Type	1	2	3	4	5	Total	Change	Roll-In (%)
**1**	476.24	19.57	0.07	16.1	0.08	512.06	35.81	25.62
**2**	22.67	66.31	0.02	6.44		95.44	29.13	20.84
**3**	0.07		17.16	0.97	0.32	18.52	1.36	0.97
**4**	4.85	0.002	1.19	511.86	5.29	523.19	11.33	8.11
**5**	0.67		0.69	28.63	33.96	63.95	29.99	21.46
**Total**	535.43	86.36	19.13	564.72	39.65	1245.31		
**Change**	59.19	20.06	1.97	52.86	5.7		139.78	
**Roll-Out (%)**	42.35	14.35	1.41	37.82	4.08			100

Note: Numbers 1–5 in the table represents ecosystem types, representing forest, grassland, wetland, farmland and urban, respectively. Other unutilized types are objective existing types, and there is mutual transformation among them. Therefore, the calculation of total area, total change and change rate all includes other unutilized. Other unutilized is not selected for analysis, so it is not shown in the transition matrix. Subtract the corresponding results from 1 to 5, respectively, to obtain others unutilized changes.

**Table 5:** Correlation results of ecosystem types and landscape pattern index changes in 9 intensities zone.

Item	Forest	Grassland	Wetland	Farmland	Urban	NP	PD	LPI	MPS
**Correlation**	0.969	0.962	0.524	0.922	0.513	0.756	0.756	0.989	0.752
**Ranking**	2	3	8	4	9	5	6	1	7

**Table 6:** The coupling coordination level of ecosystem changes in the 9 intensities zone from 2000 to 2018.

Time	C Value	T Value	D Value	Coordination Level	Coupling Coordination Degree
2000	0.846	0.602	0.714	8	Moderate coordination
2005	0.02	0.5	0.1	2	Serious imbalance
2010	0.369	0.346	0.358	4	Mild imbalance
2015	0.02	0.5	0.1	2	Serious imbalance
2018	0.22	0.35	0.277	3	Moderate imbalance

**Table 7:** Ranking of ecosystem type change results (county).

Type	Positive Ideal Distance D+	Negative Ideal Distance D−	Relative Proximity C	Sorting Result
**Forest**	0.213	0.182	0.461	4
**Grassland**	0.186	0.203	0.522	3
**Wetland**	0.116	0.206	0.64	2
**Farmland**	0.224	0.178	0.442	5
**Urban**	0.074	0.279	0.789	1

## Data Availability

The data set is provided by Data Center for Resources and Environmental Sciences, Chinese Academy of Sciences(RESDC). (http://www.resdc.cn, accessed on August 2020).
